# BioSearch: an in-house developed lab information management system for center of excellence in genomic medicine research

**DOI:** 10.1186/1471-2164-15-S2-P41

**Published:** 2014-04-02

**Authors:** Sajjad Karim, Mona SZ Al-Kharraz, Abdelbaset S Buhmeida, Mamdooh A Gari, Adeel GA Chaudhary, Adel M Abuzenadah, Mohammed H Al-Qahtani

**Affiliations:** 1 Center of Excellence in Genomic Medicine Research, King Fahd Medical Research Center, King Abdulaziz University, PO Box 80216, Jeddah 21589, Saudi Arabia

## Background

Databases and biobanks are developed in relation to a research question having its own strategy and specific demands on quality and annotation of the collected samples, resulting in multiple designs according to the different possible goals [1]. Translational research is highly dependent on large series of cases including high quality samples and their associated data.

## Materials and methods

We used ASP.NET as front-end tool, SQL Server Management Studio as back-end tool, and JavaScript and Ajax control toolkit for client-side purpose.

## Results

**Figure 1:**
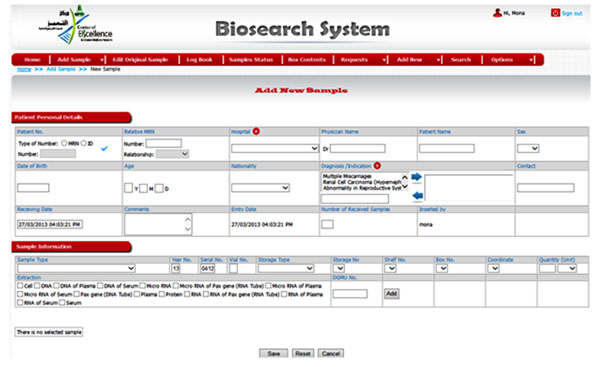
Biosearch System; an interactive database allowing valid researchers to access specimen details and request required specimen derivatives

We successfully developed an in-house Clinical Database and Biobank Management System called BioSearch, a disease-oriented general Biobank, with the goals correspond to disease biomarkers and drug target discovery through prospective and/or retrospective collections of samples and their derivates (DNA/RNA/proteins), usually associated with clinical data (Figure 1). BioSearch manages samples and clinical data of more than 5,000 patients collected between 2005 and 2013. It is composed of two well-connected units: Clinical database, a web-based database for clinical data associated with collected samples and Biobank Management System, a locally hosted database to manage the samples and their derivatives. Presently BioSearch is acting as a backbone for high-throughput genomic studies of CEGMR involved in translational research and personalized medicine.

## Conclusions

In conclusion, “BioSearch” is a platform independent highly flexible web-based user friendly system allowing clinician, researchers and biobank staff to submit, store and retrieve samples and their associated clinical information. In future, we plan to extend its capabilities by implementing new plug-in devoted to experimental research data and bioinformatics for data analysis.

*Authors would like to acknowledge the KACST*, *Riyadh*, *Saudi Arabia* (*Project ID: 10-BIO1258-03 and 10BIO1073-03*) *for funding the research.*
